# Plasma Neutrophil Gelatinase-Associated Lipocalin and Predicting Clinically Relevant Worsening Renal Function in Acute Heart Failure

**DOI:** 10.3390/ijms18071470

**Published:** 2017-07-08

**Authors:** Kevin Damman, Mattia A.E. Valente, Dirk J. van Veldhuisen, John G.F. Cleland, Christopher M. O’Connor, Marco Metra, Piotr Ponikowski, Gad Cotter, Beth Davison, Michael M. Givertz, Daniel M. Bloomfield, Hans L. Hillege, Adriaan A. Voors

**Affiliations:** 1 Department of Cardiology, University Medical Center Groningen, University of Groningen, Groningen 9712, The Netherlands; m.a.e.valente@umcg.nl (M.A.E.V.); d.j.van.veldhuisen@umcg.nl (D.J.v.V.); h.hillege@umcg.nl (H.L.H.); A.a.voors@umcg.nl (A.A.V.); 2 Imperial College London, London SW7 2AZ, UK; j.cleland@imperial.ac.uk; 3 Inova Heart and Vascular Institute, Falls Church, VA 22042, USA; Christopher.Oconnor@inova.org; 4 University of Brescia, Brescia 25121, Italy; metramarco@libero.it; 5 Medical University, Clinical Military Hospital, Wroclaw 50-981, Poland; piotrponikowski@4wsk.pl; 6 Momentum Research, Durham, NC 27707, USA; GadCotter@momentum-research.com (G.C.); bethdavison@momentum-research.com (B.D.); 7 Brigham and Women’s Hospital, Boston, MA 02115, USA; MGIVERTZ@PARTNERS.ORG; 8 Merck Research Laboratories, Rahway, NJ 07065, USA; dbloomfield1@gmail.com; 9 Department of Epidemiology, University Medical Center Groningen, University of Groningen, Groningen 9712, The Netherlands

**Keywords:** heart failure, Neutrophil Gelatinase-Associated Lipocalin, NGAL, creatinine, worsening renal function, acute heart failure

## Abstract

The aim of this study was to evaluate the ability of Neutrophil Gelatinase-Associated Lipocalin (NGAL) to predict clinically relevant worsening renal function (WRF) in acute heart failure (AHF). Plasma NGAL and serum creatinine changes during the first 4 days of admission were investigated in 1447 patients hospitalized for AHF and enrolled in the Placebo-Controlled Randomized Study of the Selective A_1_Adenosine Receptor Antagonist Rolofylline for Patients Hospitalized with Acute Decompensated Heart Failure and Volume Overload to Assess Treatment Effect on Congestion and Renal Function (PROTECT) study. WRF was defined as serum creatinine rise ≥ 0.3 mg/dL through day 4. Biomarker patterns were described using linear mixed models. WRF developed in 325 patients (22%). Plasma NGAL did not rise earlier than creatinine in patients with WRF. After multivariable adjustment, baseline plasma NGAL, but not creatinine, predicted WRF. AUCs for WRF prediction were modest (<0.60) for all models. NGAL did not independently predict death or rehospitalization (*p* = n.s.). Patients with WRF and high baseline plasma NGAL had a greater risk of death, and renal or cardiovascular rehospitalization by 60 days than patients with WRF and a low baseline plasma NGAL (p for interaction = 0.024). A rise in plasma NGAL after baseline was associated with a worse outcome in patients with WRF, but not in patients without WRF (*p* = 0.007). On the basis of these results, plasma NGAL does not provide additional, clinically relevant information about the occurrence of WRF in patients with AHF.

## 1. Introduction

Worsening renal function (WRF) during hospitalization for acute heart failure (AHF) is associated with poorer outcome. However, some studies suggest that transient WRF during treatment for AHF may not be harmful, and may even reflect a better therapeutic response [1,2,3]. We recently showed that patients with AHF and a good diuretic response had a higher incidence of WRF but better outcomes [4,5]. The cause of WRF appears to be an important factor for determining risk related to WRF. Early identification of patients at risk of WRF, as well as a robust definition and better understanding of its cause and consequences, may improve risk stratification. Novel biomarkers may play a role in achieving this goal.

Neutrophil Gelatinase Associated Lipocalin (NGAL), a 25 kDa member of the Lipocalin family expressed by the renal tubular epithelium, is released into both urine and blood in response to tubular injury. Higher plasma NGAL has been associated with poorer clinical outcomes in AHF [6,7,8]. A number of small studies showed conflicting findings on the potential value of plasma NGAL as an early marker of WRF [6,9,10,11,12,13,14,15]. For instance, in 207 patients with AHF, neither serum creatinine nor NGAL was able to accurately predict WRF [10]. In sharp contrast, in another study in 119 AHF patients, NGAL (below a certain cutoff value) had a 100% negative predictive value for the prediction of WRF [13]. A recent study evaluating NGAL as a predictor of acute kidney injury (AKI) showed similar results for NGAL, compared with serum creatinine, in the ability to predict AKI [16]. In the present study, we aimed to establish the value of plasma NGAL as an early predictor of WRF, and as a discriminator between WRF with a good and a poor prognosis.

## 2. Results

Patient characteristics for patients with and without WRF, stratified by survival, are presented in Table 1. Patients who developed WRF during the first four days (*n* = 325; 22%) had a higher Left Ventricular Ejection Fraction (LVEF), higher systolic blood pressure, less edema, worse baseline renal function, higher NGAL levels, lower hemoglobin, and more anemia (all *p* < 0.05). Profiles for survivors at 180 days versus patients who died were similar, regardless of whether WRF developed; patients who died had a lower ejection fraction, lower blood pressure, worse renal function, reflected by higher Blood Urea Nitrogen (BUN), and plasma concentrations of creatinine and NGAL (all *p* < 0.05). Tables S1 and S2 present baseline characteristics, by tertiles, of baseline serum creatinine and plasma NGAL. Higher levels were associated with more advanced age and more co-morbidity. Plasma concentrations of NGAL, correlated with serum creatinine (Spearman’s rho 0.58 at baseline and 0.60 at day 4, *p* < 0.001), estimated GFR (Spearman’s rho −0.60 at baseline and −0.62 at day 4, *p* < 0.001) and BUN (Spearman’s rho 0.52 at baseline and 0.54 at day 4, *p* < 0.001), and modestly with CRP (Spearman’s rho 0.12 at day 1, 0.13 at day 4, *p* < 0.001).

### 2.1. Neutrophil Gelatinase-Associated Lipocalin (NGAL) and Worsening Renal Function (WRF)

**Figure 1:**
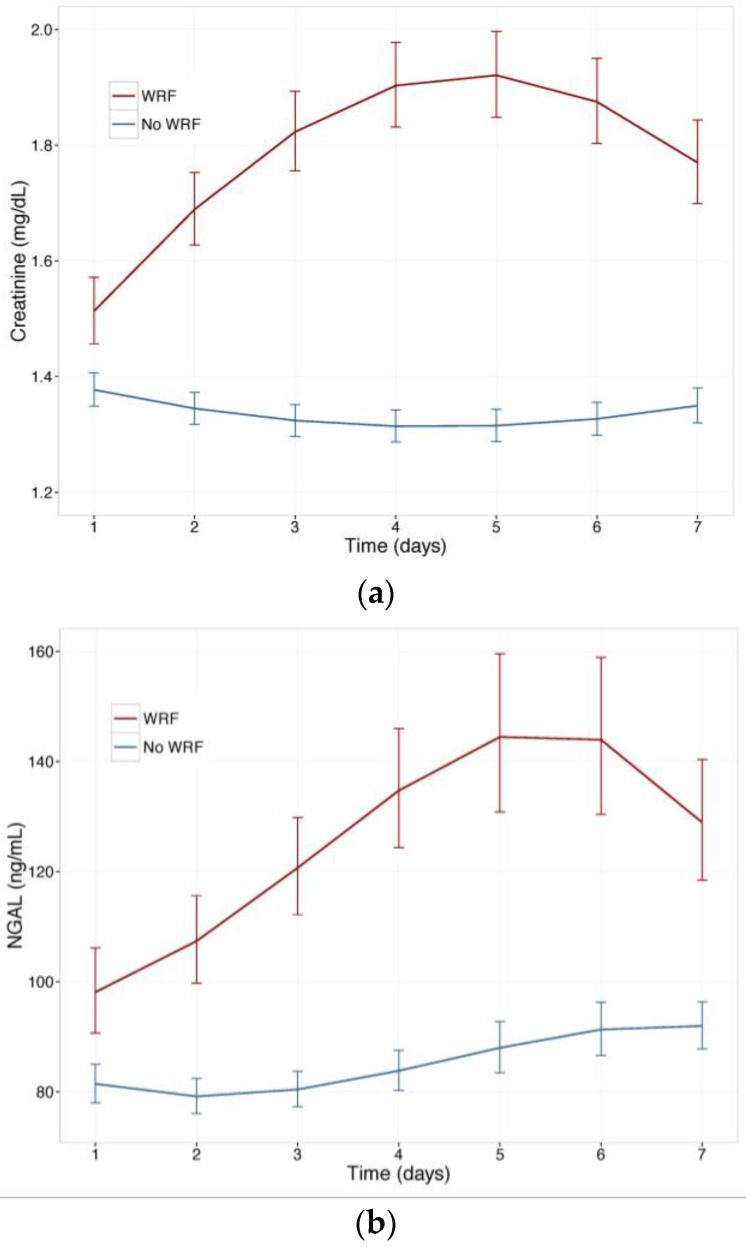
(**a**) Change in serum creatinine in patients with and without WRF; (**b**) Change in plasma Neutrophil Gelatinase-Associated Lipocalin (NGAL) in patients with and without WRF. Least square means, with 95% confidence intervals, WRF: worsening renal function, defined as a creatinine increase of ≥0.3 mg/dL by day 4.

**Figure 2:**
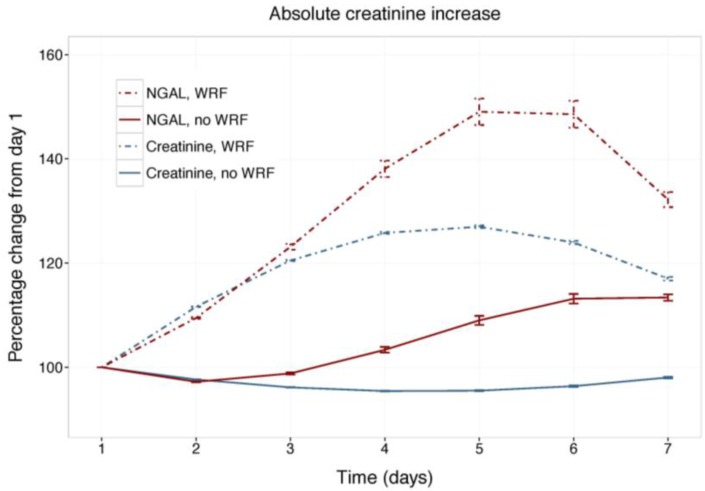
Relative changes in serum creatinine and NGAL. Least square means with 95% confidence intervals. WRF: worsening renal function, defined as creatinine increase of ≥0.3 mg/dL by day 4.

Figure 1 displays changes in serum creatinine and NGAL during the first week of admission in patients with and without WRF, adjusted for study treatment. Biomarker changes for alternative WRF definitions are presented as supplementary Figures S1 and S2, and display similar patterns. Serum creatinine and plasma NGAL trajectories differed significantly between patients with and without WRF for all definitions (*p* for interaction with time <0.001), and rolofylline treatment had no effect (*p* = n.s.). Figure 2 shows the relative creatinine and plasma NGAL changes over the first 7 days in patients with and without WRF. In patients who developed WRF, NGAL levels did not rise significantly sooner than creatinine levels; both markers increased in parallel over the first 2 days (*p* for difference n.s.), with NGAL rising further than creatinine over the course of 7 days, while displaying greater variability (*p* < 0.05). Patterns were similar for alternative definitions of WRF (Figures S3 and S4).

In ROC curve analyses, WRF proved difficult to predict well, with modest AUC values. Baseline and day 2 NGAL and creatinine were similarly predictive of WRF, while a non-diagnostic creatinine change on day 2 was a much stronger predictor of WRF than NGAL change on day 2 (Table 2).

In sensitivity analyses, predictive value for other WRF definitions/cut-offs showed similar patterns (Tables S3 and S4). Baseline plasma NGAL was independently predictive of WRF in a multivariable model (Table 3), while serum creatinine was not. NGAL contributed significantly to improving WRF prediction (AUC 0.648 vs. 0.635 for model with vs. without NGAL, *p* = 0.002). In sensitivity analyses, multivariable models for other WRF cut-offs consistently included NGAL, which significantly improved model discrimination in all models (*p* < 0.05), but not creatinine. However, these small improvements are likely not clinically relevant.

### 2.2. NGAL and Clinically Relevant WRF

**Figure 3:**
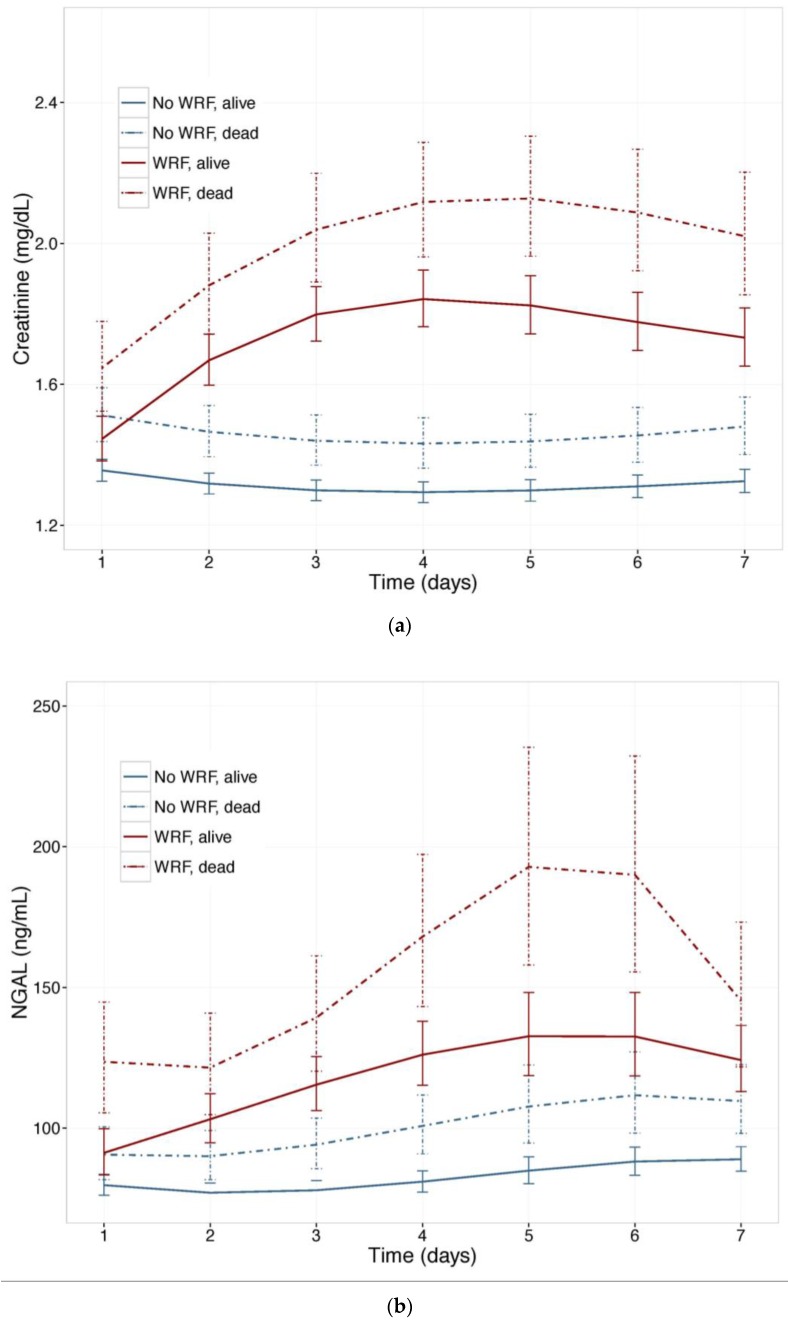
(**a**) Changes in serum Creatinine; and (**b**) NGAL in patients with and without worsening renal function, by vital status, at 180 days. Least square means with 95% confidence intervals. WRF: worsening renal function, defined as a creatinine increase of ≥0.3 mg/dL by day 4.

To investigate the value of NGAL for distinguishing between WRF with good and poor outcomes, we examined NGAL and creatinine trajectories (Figure 3) in patients who experienced WRF (or not) who had died, or were alive, after 180 days. Baseline plasma NGAL was higher and rose further in patients who died compared to survivors, and was higher in patients with WRF, irrespective of outcome. The pattern was similar when the 60-day endpoint was examined. In mutually adjusted models, including NGAL and Creatinine, NGAL was a stronger predictor of WRF with a poor outcome, and improved the model when added to creatinine, showing consistently higher Goodness of Fit (Table 4).

### 2.3. NGAL, WRF, and Clinical Outcome

In Cox models, baseline serum creatinine and plasma NGAL were invariably associated with both 180-day mortality and the 60-day composite (all *p* < 0.001), although this did not persist following multivariable adjustment (all *p* = n.s.). WRF was independently associated with both endpoints (multivariable HR for 180-day mortality: 1.45, 95% CI 1.12–1.88, *p* = 0.004; multivariable HR for the 60-day composite: 1.27, 95% CI 1.01–1.59, *p* = 0.04); sensitivity analyses with absolute creatinine change and other definitions for WRF showed similar performance (data not shown).

In patients with a rise in serum creatinine, higher baseline plasma NGAL (*p* for interaction 0.024)—but not higher baseline serum creatinine (*p* for interaction = 0.464)—posed a significant, relatively greater risk of reaching the 60-day composite endpoint. Supplementary Figure S5 displays the multivariable hazard ratios for the 60-day composite endpoint for the continuous interactions between creatinine change, and baseline values of creatinine or NGAL. The continuous hazard functions for creatinine change, stratified by either baseline creatinine levels (first panel) or baseline NGAL levels (second panel), illustrate the interaction between creatinine change and baseline biomarker levels. Patterns were similar for the 180-day mortality endpoint, but no interactions reached significance.

**Figure 4:**
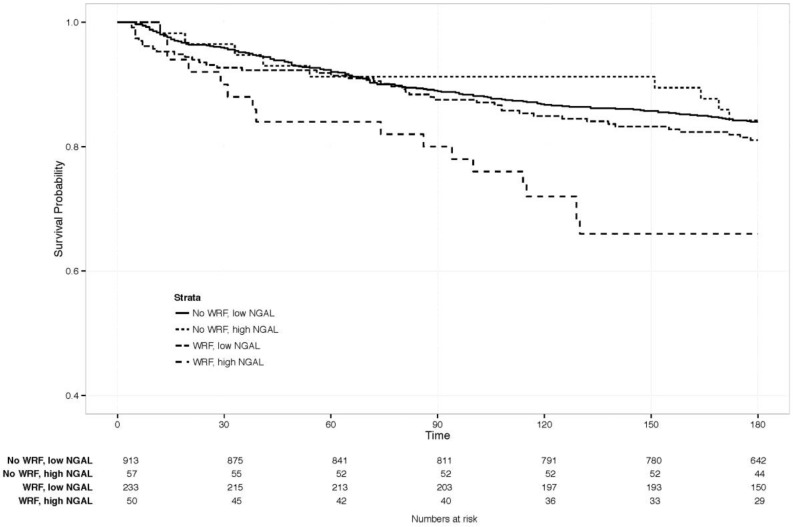
180-day survival in patients with vs. without WRF, high vs. low NGAL change. Creat: creatinine. NGAL: Neutrophil Gelatinase-Associated Lipocalin. High vs. low, defined as an NGAL increase of ≥1 standard deviation (≥88 ng/mL increase).

The clinical value of changes in NGAL, combined with WRF, was examined by comparing clinical outcomes between patients with and without WRF, and with a similar rise in NGAL on day 4. This was defined as an increase of ≥1 SD (≥88 ng/mL) in NGAL (*n* = 83), as the SD for creatinine change by day 4 was about 0.3 mg/dL, resembling the definition of WRF. The Kaplan-Meier curve is displayed in Figure 4, showing a significantly increased risk of mortality, only if both markers rose significantly (*p*-value for WRF with NGAL increase ≥88 ng/mL versus the other three groups = 0.007). Patients with ≥1 SD rise in NGAL had significantly higher creatinine at baseline than those who did not (*p* < 0.05), but similar levels, irrespective of whether WRF developed (1.8 vs. 1.7 mg/dL with vs. without WRF, *p* = 0.59). Baseline characteristics in this small subgroup did not differ significantly based on WRF status.

## 3. Discussion

We examined the value of NGAL for predicting clinically relevant WRF and outcomes in 1447 patients admitted with AHF (to our knowledge, the largest cohort of AHF patients with available serial plasma NGAL measurements). WRF was common, occurring in 22% of patients during the first four days of admission. Patients who developed WRF were more likely to have poor renal function at baseline, although only NGAL levels—but not creatinine—were independently associated with the development of WRF. We found no indication that plasma NGAL rises earlier than creatinine in AHF patients who develop WRF; both markers rose in tandem over the first two days of admission. Although NGAL showed statistically significant incremental value for predicting WRF, no combination of markers performed particularly well, with AUCs below 0.60. While high levels of both NGAL and creatinine at baseline were associated with mortality and rehospitalization, neither was independently predictive after adjustment for clinical covariates.

### 3.1. Prediction of WRF

NGAL has been identified as a powerful early predictor of WRF in a number of different clinical settings [17,18,19,20,21,22,23,24,25], although the data in AHF are conflicting [6,7,10,11,12,13,14,15,26]. Similarly to Breidthardt et al. [10], we found modest predictive ability for plasma NGAL, which provided minimal improvement on top of creatinine for predicting WRF. Our data further confirm findings by the prospective biomarker study Acute Kidney Injury Neutrophil Gelatinase-Associated Lipocalin Evaluation of Symptomatic Heart Failure Study (AKINESIS) that recently showed similar results—NGAL was not a more sensitive predictor of AKI in patients admitted with acute HF [16]. Whereas AKINESIS only assessed severe and sustained increases in creatinine, more closely resembling acute kidney injury, we evaluated the much more common used definitions of WRF. Furthermore, our study not only provides data on inhospital changes in plasma NGAL, but also long term outcomes, whereas in AKINESIS only inhospital events were evaluated. In the end, considering the poor performance of both markers for AKI/WRF prediction in both AKINESIS and the present study, the clinical relevance of the associations is debatable at best.

One potential issue is the self-fulfilling nature of predicting a rise in creatinine, using creatinine. Interestingly, we found that baseline NGAL values—but not baseline creatinine values—predicted WRF in multivariable models. Regardless, the hypothesis that NGAL rises earlier than creatinine does not hold true in this AHF cohort, as illustrated by the estimated trajectories corrected for study treatment. There are several potential explanations for the lack of an early rise in plasma NGAL, and thus the poor prognostic accuracy for WRF in AHF. First, plasma NGAL—in contrast with urinary NGAL—may not be a particularly appropriate tubular marker; it is strongly related to glomerular filtration rate, as reflected by its correlation with GFR and creatinine, and also involved in iron scavenging and immune response, as indicated by the correlation with markers such as CRP and markers of anemia [27,28]. Shrestha et al noted strong correlations between urinary NGAL and measures for natriuresis and response to diuretics, while plasma NGAL only correlated well with GFR, though both predicted WRF [14]. Second, there are multiple mechanisms for WRF in AHF patients. For example, true AKI (resulting in tubular damage), with substantial and rapid loss of function and decreased urine output is probably not comparable to the kind of WRF studied extensively in AHF. In the clinical context of AHF, changes in renal function may be driven more by hemodynamic and neurohormonal (mal) adaptation and drug effects than the (hypoxic) kidney injury common in intensive care or post-surgical settings; Dupont et al. showed that despite a relatively high incidence of AKI, defined based on creatinine increases, tubular injury was relatively uncommon in a small, prospective study of 141 AHF patients [26]. Third, in contrast with studies in post-surgical or post-intervention patients [17,18,19,24,25,29], the timing of renal injury is often unclear in AHF, and its pre-hospital course may vary significantly, and may include undetected WRF. Pre-admission worsening congestion and intensification of diuretic therapy may have already triggered progressive renal impairment in the patients in our study—all of whom had at least a brief history of heart failure. Fourth, there is ongoing debate regarding the best measure for renal function and injury; a definition of WRF, based on a more “pure” marker, such as cystatin C or measured GFR, may have yielded very different results.

However, it is noteworthy that our additional analysis of clinically relevant WRF (that is, WRF associated with poor clinical outcome) showed patients with WRF (and an adverse outcome had much higher NGAL levels).

### 3.2. NGAL, Creatinine and Outcome

Impaired and worsening renal function are established risk markers in heart failure [30]. Data on the prognostic value of NGAL is mixed, with many [7,8,12,31,32,33,34]—but not all [35]—studies in both chronic and AHF reporting prognostic value, though correction for potential confounders varies greatly. Givertz et al. previously reported on the prognostic value of various renal markers in PROTECT, concluding that creatinine change and baseline BUN were strong predictors of outcome [36]. Additionally, our analyses show that NGAL modulates the risk of outcomes associated with creatinine change, conferring a greater relative risk to patients with higher NGAL levels with a creatinine increase, but not to patients without. This effect is independent of baseline creatinine. Furthermore, a large increase in NGAL during admission conferred an additional risk of death (only in patients with WRF). Thus, while plasma NGAL levels appear to largely reflect GFR (and thus creatinine), they do have some incremental value for assessing the risk associated with WRF, and could help discriminate between higher and lower risk WRF.

### 3.3. Clinical Perspectives

Identifying patients at high risk of developing renal dysfunction and poor outcomes remains a challenge in AHF. Coupled with a lack of effective therapeutic options, this poses a problem for clinicians. Biomarkers such as NGAL can be used as diagnostic or prognostic tools, though their application requires careful and thorough evaluation. Despite the extensive but conflicting literature on plasma NGAL, our analyses in this very large group of well-characterized AHF patients, together with findings from AKINESIS, indicate fairly poor accuracy for predicting WRF.

Plasma NGAL was not independently prognostic for death or rehospitalization. Our findings suggest elevated baseline NGAL levels, and large increases in NGAL do confer additional risk for patients who develop WRF (although the clinical relevance of these findings—given the lack of independent prognostic value for NGAL, the small numbers of patients with NGAL increases, and the post-hoc nature of our analyses—remains to be established).

### 3.4. Limitations

Due to the retrospective nature of this study, our results should be considered hypothesis-generating, and should therefore be interpreted cautiously. Furthermore, NGAL was measured using frozen samples, which may have affected data quality. No urine was collected, so the performance of urinary NGAL could not be compared that of plasma NGAL, and may have shown very different patterns and results.

## 4. Materials and Methods 

### 4.1. Study Design and Population

This is a *post hoc* analysis of the Placebo-controlled Randomized Study of the Selective A1 Adenosine Receptor Antagonist Rolofylline for Patients Hospitalized with Acute Heart Failure and Volume Overload to Assess Treatment Effect on Congestion and Renal FuncTion (PROTECT) trial, a randomized, double-blind, placebo-controlled, multi-center study that enrolled 2033 patients admitted for acute decompensated heart failure, randomized 2:1 to rolofylline, with neutral overall results. Study design, inclusion, and exclusion criteria and results have been published previously [37,38,39]. The trial was approved by all local Ethics Committees and conducted in accordance with the Declaration of Helsinki (NCT00328692 and NCT00354458). All patients provided written informed consent. Of the patients who remained hospitalized for at least 4 days (*n* = 1681), those with available NGAL and creatinine values at baseline (*n* = 1470), and at least one follow-up measurement for each marker during the first 4 days, were included in the analysis, resulting in a study population of 1447 patients. Patients who had already developed WRF by day 2 (*n* = 101) were excluded from analyses of the effects of biomarker levels and changes on day 2.

### 4.2. Procedures and Definitions

Heart failure signs and symptoms, serum creatinine, and other hematologic and biochemical markers, were assessed daily from baseline (day 1) until discharge or day 6 and on day 7, as dictated by study protocol [37]. Plasma NGAL levels were measured in frozen plasma samples collected on the same days and stored at −80 °C. Measurements were performed by Alere Inc. (San Diego, CA, USA) using sandwich enzyme-linked immunosorbent assays (ELISA) on a microtiter plate. The estimated glomerular filtration rate (GFR) was calculated using the simplified modification of diet in renal disease (MDRD) study equation.

### 4.3. Endpoints

This study examined WRF occurring during the first 4 days of hospitalization, defined as a creatinine increase of ≥0.3 mg/dL (26.5 µmol/L), at any time between day 1 (baseline) and day 4, or initiation of hemofiltration as it was defined in the main study. Sensitivity analyses were performed with other definitions, including absolute creatinine, a relative creatinine increase of ≥25%, a combined increase of ≥0.3 mg/dL and ≥25%, and various cut-offs.

The prognostic value of plasma NGAL for distinguishing between WRF and good vs. poor prognosis was examined for adjudicated endpoints of 180-day mortality and a composite of 60-day death or renal or cardiovascular rehospitalization. WRF with good vs. poor outcome was defined based on whether patients experienced either of the clinical endpoints.

### 4.4. Statistical Methods

Continuous data are presented as mean ± SD if normally distributed, or median [interquartile range] if not. Group comparisons were performed using Student’s *t*-test, ANOVA, Wilcoxon, or Kruskall-Wallis tests, as appropriate. Differences between relative changes in biomarkers were assessed using paired Wilxocon rank sum tests. Correlations between biomarkers were evaluated using Spearman’s rank correlation. Missing data were assumed to be missing at random, and no imputations were performed.

Changes in serial biomarker measurements were evaluated using random slope, random intercept linear mixed-effects models, adjusted for study treatment. A mixed-effects model is a hierarchical regression model that includes fixed and random (subject-specific) effects, allowing for within-subject correlation between repeated measurements. Both NGAL and creatinine were log-transformed for modeling. Model selection was based on combined assessment of likelihood ratio tests of nested models for selection of random effects, and of Bayesian and Akaike’s information criteria (measures for model fit, lower is better) for selection of fixed effects, following graphical exploration of the data. Best fit was obtained using a second order polynomial (quadratic) time transformation for creatinine and third order polynomial (cubic) time transformation for NGAL, for both fixed and random effects.

Receiver Operator Characteristic (ROC) curve analyses and multivariable logistic regression were performed to evaluate predictors of WRF, and added value in multivariable models was assessed using likelihood ratio tests of nested models. Multivariable models were constructed via backward elimination of candidate covariates with a univariable association at *p* < 0.1, with a *p* for retention of 0.05.

Kaplan Meier survival analyses were performed to examine group associations with the mortality and composite endpoints. Outcomes between groups were compared with log-rank tests. Cox proportional hazards regression was performed to evaluate univariable and multivariable associations with 180-day mortality and the 60-day composite, adjusting for covariates from a previously published prognostic model—age, creatinine, BUN, systolic blood pressure, edema, previous hospitalization for heart failure, serum albumin, and serum sodium [40]. Multiple fractional polynomials were used to check for non-linearity in survival analyses. Interactions were investigated graphically. Proportionality of hazards assumptions were evaluated graphically and tested statistically. A two-tailed *p*-value of 0.05 was considered statistically significant. All analyses were performed using R: A Language and Environment for Statistical Computing, version 3.1.0 (R Foundation for Statistical Computing, Vienna, Austria) and Stata, version 11.2 (College Station, TX, USA).

## 5. Conclusions

Plasma NGAL was not independently predictive of poor outcome, though serial plasma NGAL levels provide some additional information for the prediction of clinically significant WRF in patients with AHF. However, plasma NGAL levels did not rise earlier than creatinine in patients who developed WRF, and both NGAL and creatinine were similarly modest predictors of WRF.

## Figures and Tables

**Table 1:** Baseline characteristics for patients with and without worsening renal function (WRF), and by vital status, at day 180.

Categories	No WRF, Alive	No WRF, Dead	*p* *	No WRF, Total	WRF, Alive	WRF, Dead	p *	WRF, Total	*p* **
	(*n =* 936)	(*n =* 186)		(*n =* 1122)	(*n =* 247)	(*n =* 78)		(*n =* 325)	
**Demographics**									
Sex (% Male)	64 (599)	68.3 (127)	0.302	64.7 (726)	69.6 (172)	71.8 (56)	0.825	70.2 (228)	0.079
Age (years)	69.6 ± 11.6	72.4 ± 10.7	0.001	70 ± 11.5	71.1 ± 10.3	71.9 ± 10.7	0.578	71.3 ± 10.4	0.058
BMI (kg/m^2^)	28.8 ± 6	27.8 ± 5.7	0.032	28.6 ± 6	29.1 ± 5.8	28.3 ± 6	0.311	28.9 ± 5.9	0.516
LVEF (% (*n*))	32.7 ± 12.8	28.6 ± 11	0.001	31.9 ± 12.5	36.5 ± 13.9	30.5 ± 13.4	0.021	34.9 ± 14	0.020
Systolic Blood Pressure (mmHg)	125.7 ± 17.3	117.8 ± 17.2	<0.001	124.4 ± 17.6	129.2 ± 16.6	120.1 ± 16.4	<0.001	127 ± 17	0.015
Diastolic Blood Pressure (mmHg)	74.8 ± 11.5	71 ± 10.7	<0.001	74.1 ± 11.5	76.9 ± 12.1	71.2 ± 10.8	<0.001	75.5 ± 12	0.060
Heart Rate (beats/min)	81.6 ± 16.4	80.3 ± 15.1	0.286	81.4 ± 16.2	80.3 ± 14.9	78.8 ± 14.8	0.452	79.9 ± 14.9	0.124
Rolofylline administration (% (*n*))	65.2 (610)	69.4 (129)	0.310	65.9 (739)	72.5 (179)	65.4 (51)	0.291	70.8 (230)	0.112
**Clinical Profile**									
Orthopnea (% (*n*))	96.6 (896)	94.6 (175)	0.287	96.2 (1071)	95.1 (233)	100 (78)	0.099	96.3 (311)	1.000
Rales (% (*n*))	63.5 (594)	61.3 (114)	0.621	63.2 (708)	64.8 (160)	65.4 (51)	1.000	64.9 (211)	0.605
Edema (% (*n*))	71.2 (666)	71 (132)	1.000	71.1 (798)	63.6 (157)	60.3 (47)	0.695	62.8 (204)	0.005
Jugular venous pressure (% (*n*))	41.2 (344)	49.4 (81)	0.064	42.5 (425)	40.3 (87)	43.7 (31)	0.716	41.1 (118)	0.716
**Medical History**									
Hypertension (% (*n*))	80.1 (750)	79 (147)	0.810	79.9 (897)	79.8 (197)	74.4 (58)	0.394	78.5 (255)	0.612
Diabetes Mellitus (% (*n*))	44.9 (420)	45.7 (85)	0.899	45 (505)	44.5 (110)	41.6 (32)	0.743	43.8 (142)	0.754
Hypercholesterolemia (% (*n*))	45.7 (427)	42.5 (79)	0.472	45.1 (506)	51.4 (127)	48.7 (38)	0.775	50.8 (165)	0.084
Smoking (% (*n*))	18.6 (174)	19.5 (36)	0.867	18.8 (210)	14.2 (35)	20.5 (16)	0.250	15.7 (51)	0.247
Ischemic Heart Disease (% (*n*))	69.3 (648)	74.6 (138)	0.177	70.2 (786)	69.2 (171)	73.1 (57)	0.613	70.2 (228)	1.000
Myocardial Infarction (% (*n*))	49 (458)	54.3 (100)	0.216	49.9 (558)	48.2 (119)	52.6 (41)	0.585	49.2 (160)	0.879
PCI (% (*n*))	21.9 (203)	26.8 (49)	0.182	22.7 (252)	22.3 (55)	23.4 (18)	0.962	22.5 (73)	1.000
CABG (% (*n*))	18.6 (172)	21.9 (40)	0.349	19.1 (212)	21.9 (54)	25.6 (20)	0.590	22.8 (74)	0.168
Peripheral Vascular Disease (% (*n*))	10.2 (95)	15.1 (28)	0.069	11 (123)	11 (27)	14.3 (11)	0.559	11.8 (38)	0.769
Atrial Fibrillation (% (*n*))	55.6 (518)	57 (106)	0.797	55.9 (624)	55.1 (136)	50 (39)	0.515	53.8 (175)	0.561
NYHA Class			0.426				0.059		0.578
I–II	16.6 (155)	13.4 (25)		16 (180)	19.8 (49)	10.3 (8)		17.5 (57)	
III	44.9 (420)	49.5 (92)		45.6 (512)	45.7 (113)	59 (46)		48.9 (159)	
IV	32.8 (307)	31.7 (59)		32.6 (366)	31.6 (78)	26.9 (21)		30.5 (99)	
ICD therapy (% (*n*))	12.6 (118)	17.2 (32)	0.119	13.4 (150)	12.6 (31)	19.2 (15)	0.197	14.2 (46)	0.790
CRT therapy (% (*n*))	7.2 (67)	14 (26)	0.003	8.3 (93)	11.3 (28)	9 (7)	0.706	10.8 (35)	0.205
Stroke (% (*n*))	8.3 (78)	9.1 (17)	0.828	8.5 (95)	12.6 (31)	19.2 (15)	0.197	14.2 (46)	0.003
COPD (% (*n*))	19.4 (181)	21.6 (40)	0.545	19.7 (221)	18.6 (46)	21.8 (17)	0.650	19.4 (63)	0.953
**Prior Medication Use**									
ACE inhibitors or ARB (% (*n*))	76.4 (715)	69.9 (130)	0.074	75.3 (845)	76.5 (189)	71.8 (56)	0.488	75.4 (245)	1.000
Beta blockers (% (*n*))	74.7 (699)	75.3 (140)	0.939	74.8 (839)	74.5 (184)	67.9 (53)	0.323	72.9 (237)	0.547
MRA (% (*n*))	45.8 (429)	48.4 (90)	0.577	46.3 (519)	47.4 (117)	59 (46)	0.097	50.2 (163)	0.239
Calcium Antagonists (% (*n*))	12.7 (119)	8.6 (16)	0.147	12 (135)	21.9 (54)	7.7 (6)	0.008	18.5 (60)	0.004
Nitrates (% (*n*))	26.9 (252)	26.9 (50)	1.000	26.9 (302)	27.5 (68)	29.5 (23)	0.849	28 (91)	0.752
Digoxin (% (*n*))	31 (290)	31.2 (58)	1.000	31 (348)	32.4 (80)	20.5 (16)	0.063	29.5 (96)	0.660
**Laboratory Values**									
Creatinine (mg/dL)	1.3 (1.1–1.7)	1.5 (1.2–2.1)	<0.001	1.3 (1.1–1.7)	1.4 (1.2–1.8)	1.7 (1.3–2)	0.002	1.5 (1.2–1.8)	<0.001
eGFR (mL/min/1.73 m^2^)	52 (39–66)	45 (33–60)	<0.001	51 (38–65)	48 (38–62)	40 (32–51)	<0.001	46 (37–59)	<0.001
NGAL (ng/mL)	78 (50–123)	96 (58–137)	0.008	81 (52–127)	90 (56–142)	131 (72–187)	0.002	93 (58–151)	<0.001
Blood Urea Nitrogen (mg/dL)	28 (21–38)	37 (26–51)	<0.001	29 (22–40)	28 (23–38)	41 (30–55)	<0.001	31 (24–43)	0.029
Sodium (mmol/L)	140 (137–143)	138 (135–141)	<0.001	140 (137–142)	141 (138–143)	139 (136–142)	0.010	140 (138–143)	0.063
Potassium (mmol/L)	4.2 (3.9–4.6)	4.3 (3.9–4.8)	0.175	4.2 (3.9–4.6)	4.3 (4–4.7)	4.3 (3.9–4.7)	0.815	4.3 (3.9–4.7)	0.090
Hemoglobin (g/dL)	12.9 ± 2	12.7 ± 1.9	0.216	12.8 ± 2	12.5 ± 1.9	12.3 ± 1.8	0.399	12.5 ± 1.9	0.007
Anemia (% (*n*))	38.3 (314)	44.4 (75)	0.169	39.4 (389)	46.6 (102)	49.3 (34)	0.800	47 (136)	0.021
BNP (mg/dL)	1195 (815–2228)	1895 (1172–3300)	<0.001	1351 (852–2433)	1073 (718–1616)	1749 (1153–2829)	0.006	1190 (779–2078)	0.227

Abbreviations: BMI: Body Mass Index; LVEF: Left Ventricular Ejection Fraction; PCI: Percutaneous Coronary Intervention; CABG: Coronary Artery Bypass Graft; NYHA: New York Heart Association; ICD: Internal Cardiac Defibrillator; CRT: Cardiac Resynchronization Therapy; COPD: chronic obstructive pulmonary disease; ACE: Angiotensin Converting Enzyme; ARB: Aldosterone Receptor Blocker; MRA: Mineralocorticoid Receptor Antagonist; eGFR: estimated Glomerular Filtration Rate; BNP: Brain Natriuretic Peptide. Categorical variables are presented as: % (N). * *p* value for dead vs. alive, ** *p* value for WRF vs. No WRF. To convert Creatinine from mg/dL to µmol/L, multiply by 88.4.

**Table 2:** Predictive value of NGAL and Creatinine for WRF.

**Baseline Values**			
WRF definition	Creatinine AUC	NGAL AUC	*p* *
≥0.3 mg/dL increase	0.571	0.569	0.930
**Day 2 Values**			
WRF definition	Creatinine AUC	NGAL AUC	*p* *
≥0.3 mg/dL increase	0.617	0.570	0.097
**Change on Day 2**			
WRF definition	Creatinine AUC	NGAL AUC	*p* *
≥0.3 mg/dL increase	0.718	0.491	<0.001

* *p* for difference in AUC.

**Table 3:** Multivariable logistic regression for prediction of worsening renal function.

Variables	OR (95% CI)	χ^2^	*p*
Cholesterol (per SD)	1.33 (1.16–1.52)	16.26	<0.001
Hemoglobin (per SD)	0.77 (0.67–0.90)	11.45	0.001
NGAL (per SD)	1.23 (1.08–1.40)	9.79	0.002
History of Stroke	1.89 (1.25–2.83)	9.52	0.002
Male Sex	1.48 (1.10–2.01)	6.55	0.010
Albumin (per SD)	1.19 (1.03–1.38)	5.77	0.016
Rolofylline treatment	1.39 (1.04–1.87)	4.78	0.029

Abbreviations: SD: standard deviation; OR: Odds Ratio.

**Table 4:** Added value of NGAL on top of creatinine for predicting clinically relevant WRF.

WRF definition	MV Model * OR (95% CI)	χ^2^	*p-value* **	AUC	*p-value* ***
WRF and 180-day mortality ****					
≥0.3 mg/dL increase					
Creatinine	1.26 (1.02–1.55)	4.81	0.028	0.670	0.021
NGAL	1.25 (1.04–1.48)	6.52	0.011		
≥25% & ≥0.3mg/dL increase					
Creatinine	1.03 (0.78–1.33)	0.04	0.833	0.637	0.017
NGAL	1.31 (1.06–1.56)	7.53	0.006		
WRF and 60-day endpoint ****					
≥0.3 mg/dL increase					
Creatinine	1.25 (1.03–1.5)	5.36	0.021	0.656	0.001
NGAL	1.32 (1.12–1.55)	11.69	0.001		
≥25% & ≥0.3mg/dL increase					
Creatinine	1.03 (0.81–1.29)	0.05	0.821	0.633	0.005
NGAL	1.31 (1.09–1.55)	9.57	0.002		

* Multivariable logistic model, including both creatinine and NGAL; odds ratios presented per standard deviation; ** Multivariable *p* value for WRF prediction; *** Likelihood ratio test for added value of adding NGAL to a model with creatinine alone; **** Prediction of WRF with poor clinical outcome, compared to all other patients.

## Supplementary Materials

Supplementary materials can be found at http://www.mdpi.com/1422-0067/18/7/1470/s1.

## Abbreviations

AHF	Acute Heart Failure
AKI	Acute Kidney Injury
LVEF	Left ventricular ejection fraction
NGAL	Neutrophil Gelatinase-Associated Lipocalin
WRF	Worsening Renal Function
HR	Hazard Ratio
SD	Standard Deviation
