# Supplementary material for: Plasma Neutrophil Gelatinase-Associated Lipocalin and Predicting Clinically Relevant Worsening Renal Function in Acute Heart Failure
Source: Int J Mol Sci. 2017 Jul 8;18(7):1470. doi: 10.3390/ijms18071470 (PMC5535961; doi:10.3390/ijms18071470)

Table S1. Baseline characteristics across creatinine tertiles

Creatinine tertiles		1 [0.9-1.2] *(n=559)*	1.4 [1.3-1.5] *(n=445)*	2 [1.8-2.4] *(n=443)*	P-lin
**Demographics**					
Sex (% Male)		53.7 (300)	67.2 (299)	80.1 (355)	<0.001
Age (years)		69.2±12	70.9±11	71.1±10.3	0.008
BMI (kg/m2)		28.2±6.5	28.9±5.8	29.1±5.3	0.019
LVEF (% (n))		33.7±13.1	32.9±13.5	30.9±12	0.021
Systolic Blood Pressure (mmHg)		125.5±17.1	125.2±17.4	124.1±18	0.187
Diastolic Blood Pressure (mmHg)		75.2±11.4	75.1±11.7	72.8±11.6	0.001
Heart Rate (beats/min)		83±16.1	81.3±15.9	78.4±15.3	<0.001
Rolofylline administration (% (n))		63.7 (356)	68.5 (305)	69.5 (308)	0.045
**Clinical Profile**					
Orthopnea (% (n))		96.2 (532)	96.8 (428)	95.7 (422)	0.711
Rales (% (n))		66 (369)	64.7 (288)	59.3 (262)	0.031
Edema (% (n))		68 (380)	71 (316)	69.1 (306)	0.663
Jugular venous pressure (% (n))		39.7 (200)	43.6 (170)	44.1 (173)	0.168
**Medical History**					
Hypertension (% (n))		78.4 (438)	81.1 (361)	79.7 (353)	0.563
Diabetes Mellitus (% (n))		38.6 (216)	43.8 (195)	53.4 (236)	<0.001
Hypercholesterolemia (% (n))		36.7 (205)	50.2 (223)	54.9 (243)	<0.001
Smoking (% (n))		15.9 (89)	18.5 (82)	20.4 (90)	0.070
Ischemic Heart Disease (% (n))		67.9 (378)	67.6 (301)	75.6 (335)	0.010
Myocardial Infarction (% (n))		46 (256)	46.6 (207)	57.6 (255)	<0.001
PCI (% (n))		19.7 (109)	20.8 (92)	28.4 (124)	0.002
CABG (% (n))		11.9 (66)	20.8 (92)	29.2 (128)	<0.001
Peripheral Vascular Disease (% (n))		8.6 (48)	11.7 (52)	13.8 (61)	0.009
Atrial Fibrillation (% (n))		53.9 (300)	59.3 (262)	53.5 (237)	0.992
NYHA Class					<0.001
	I-II	15.7 (88)	16.2 (72)	17.4 (77)	
	III	42 (235)	47.6 (212)	50.6 (224)	
	IV	37.6 (210)	30.3 (135)	27.1 (120)	
ICD therapy (% (n))		7.5 (42)	14.6 (65)	20.1 (89)	<0.001
CRT therapy (% (n))		4.8 (27)	10.6 (47)	12.2 (54)	<0.001
Stroke (% (n))		7.2 (40)	11.2 (50)	11.5 (51)	0.017
COPD (% (n))		19 (106)	17.8 (79)	22.4 (99)	0.201
**Prior Medication Use**					
ACE inhibitors or ARB (% (n))		76.2 (426)	77.5 (345)	72 (319)	0.149
Beta blockers (% (n))		69.8 (390)	76.2 (339)	78.3 (347)	0.002
MRAs (% (n))		47.8 (267)	48.3 (215)	45.1 (200)	0.433
Calcium Antagonists (% (n))		11.3 (63)	13.3 (59)	16.5 (73)	0.017
Nitrates (% (n))		23.3 (130)	26.5 (118)	32.7 (145)	0.001
Digoxin (% (n))		36.5 (204)	30.8 (137)	23.3 (103)	<0.001
**Laboratory Values**					
eGFR (mL/min/1.73m2)		68 [61-78]	48.6 [42-55]	32 [27-39]	<0.001
NGAL (ng/ml)		57 [41-84]	81 [58-127]	137 [95-204]	<0.001
Blood Urea Nitrogen (mg/dL)		22 [18-26]	30 [25-38]	45 [36-59]	<0.001
Sodium (mmol/L)		140 [138-143]	140 [137-143]	140 [137-142]	<0.001
Potassium (mmol/L)		4.2 [3.9-4.6]	4.3 [3.9-4.6]	4.4 [3.9-4.8]	<0.001
Hemoglobin (g/dL)		13.1±1.9	12.8±1.9	12.3±2	<0.001
Anemia (% (n))		28.1 (139)	40.6 (162)	58.5 (224)	<0.001
Total Cholesterol (mmol/L)		155±42	146±46	143±45	<0.001
Triglycerides (mmol/L)		99±52	98±53	104±60	0.105
BNP (mg/dL)		1172 [775-2232]	1302 [916-2152]	1362 [811-2773]	0.180

Abbreviations: BMI: Body Mass Index; LVEF: Left Ventricular Ejection Fraction; PCI: Percutaneous Coronary Intervention; CABG: Coronary Artery Bypass Graft NYHA: New York Heart Association; ICD: Internal Cardiac Defibrillator; CRT: Cardiac Resynchronization Therapy; COPD: chronic obstructive pulmonary disease; ACE: Angiotensin Converting Enzyme ; ARB: Aldosterone Receptor Blocker; MRA: Mineralocorticoid Receptor Antagonist; eGFR: estimated Glomerular Filtration Rate; BNP: Brain Natriuretic Peptide. Categorical variables are presented as: % (N).

P value: Non-parametric trend tests for categorical variables, and univariable generalized linear models with polynomial contrasts for continuous variables were used to examine trends across categories.

Table S2. Baseline characteristics across NGAL tertiles

NGAL Tertiles	44 [35-53] *(n=478)*	83 [72-94] *(n=477)*	166 [132-223] *(n=492)*	P-lin
**Demographics**				
Sex (% Male)	64.6 (309)	66.9 (319)	66.3 (326)	0.599
Age (years)	67.5±12	70.7±11.1	72.7±9.9	<0.001
BMI (kg/m2)	28.5±6.6	28.6±5.8	29±5.5	0.238
LVEF (% (n))	31.9±12.3	31.7±12.9	33.9±13.4	0.101
Systolic Blood Pressure (mmHg)	124.9±17	124.4±17.5	125.6±17.8	0.521
Diastolic Blood Pressure (mmHg)	75.8±11.3	74.6±11.7	73±11.7	<0.001
Heart Rate (beats/min)	83.8±16.4	80.8±16.1	78.7±14.9	<0.001
Rolofylline administration (% (n))	68 (325)	65.4 (312)	67.5 (332)	0.871
**Clinical Profile**				
Orthopnea (% (n))	96.2 (456)	96 (453)	96.5 (473)	0.786
Rales (% (n))	67.2 (321)	60.7 (289)	62.8 (309)	0.163
Edema (% (n))	69 (330)	70 (334)	68.7 (338)	0.906
Jugular venous pressure (% (n))	43.6 (185)	39.6 (168)	43.4 (190)	0.950
**Medical History**				
Hypertension (% (n))	77.4 (370)	78.6 (375)	82.7 (407)	0.039
Diabetes Mellitus (% (n))	35.8 (171)	45.7 (218)	52.5 (258)	<0.001
Hypercholesterolemia (% (n))	41.2 (197)	45.4 (216)	52.4 (258)	<0.001
Smoking (% (n))	18.4 (88)	15.8 (75)	20 (98)	0.533
Ischemic Heart Disease (% (n))	67.3 (321)	69.8 (333)	73.3 (360)	0.040
Myocardial Infarction (% (n))	47.9 (228)	48.7 (232)	52.5 (258)	0.147
PCI (% (n))	19 (90)	23.8 (113)	25.2 (122)	0.024
CABG (% (n))	14.2 (67)	20.1 (95)	25.4 (124)	<0.001
Peripheral Vascular Disease (% (n))	9.2 (44)	10.5 (50)	13.7 (67)	0.027
Atrial Fibrillation (% (n))	54.1 (256)	55.6 (265)	56.5 (278)	0.457
NYHA Class				0.081
I-II	15.3 (73)	15.5 (74)	18.3 (90)	
III	43.5 (208)	46.8 (223)	48.8 (240)	
IV	36.2 (173)	33.5 (160)	26.8 (132)	
ICD therapy (% (n))	10.9 (52)	14 (67)	15.7 (77)	0.031
CRT therapy (% (n))	8.6 (41)	9.2 (44)	8.8 (43)	0.931
Stroke (% (n))	9.6 (46)	7.5 (36)	12 (59)	0.208
COPD (% (n))	18.4 (88)	20.5 (98)	20 (98)	0.558
**Prior Medication Use**				
ACE inhibitors or ARB (% (n))	77.4 (370)	75.9 (362)	72.8 (358)	0.093
Beta blockers (% (n))	72.8 (348)	74.4 (355)	75.8 (373)	0.283
MRAs (% (n))	50.2 (240)	47.4 (226)	43.9 (216)	0.049
Calcium Antagonists (% (n))	9 (43)	11.9 (57)	19.3 (95)	<0.001
Nitrates (% (n))	23.8 (114)	26.8 (128)	30.7 (151)	0.017
Digoxin (% (n))	35.1 (168)	31.2 (149)	25.8 (127)	0.002
**Laboratory Values**				
Creatinine (mg/dL)	1.1 [0.9-1.3]	1.4 [1.1-1.7]	1.8 [1.4-2.3]	<0.001
eGFR (mL/min/1.73m2)	64 [52-75]	50 [40-63]	37 [29-46]	<0.001
Blood Urea Nitrogen (mg/dL)	23 [18-30]	29 [22-38]	40 [30-54]	<0.001
Sodium (mmol/L)	140 [137-143]	140 [137-143]	140 [137-142]	0.043
Potassium (mmol/L)	4.2 [3.8-4.5]	4.2 [3.9-4.6]	4.4 [4-4.8]	<0.001
Hemoglobin (g/dL)	13.4±1.9	12.7±1.9	12.2±1.9	<0.001
Anemia (% (n))	30.5 (129)	41.3 (173)	51.4 (223)	<0.001
Total Cholesterol (mmol/L)	153±43	146±47	146±44	0.015
Triglycerides (mmol/L)	97±51	99±56	105±57	0.015
BNP (mg/dL)	1265 [794-2276]	1216 [868-2238]	1340 [864-2336]	0.605

Abbreviations: BMI: Body Mass Index; LVEF: Left Ventricular Ejection Fraction; PCI: Percutaneous Coronary Intervention; CABG: Coronary Artery Bypass Graft NYHA: New York Heart Association; ICD: Internal Cardiac Defibrillator; CRT: Cardiac Resynchronization Therapy; COPD: chronic obstructive pulmonary disease; ACE: Angiotensin Converting Enzyme ; ARB: Aldosterone Receptor Blocker; MRA: Mineralocorticoid Receptor Antagonist; eGFR: estimated Glomerular Filtration Rate; BNP: Brain Natriuretic Peptide. Categorical variables are presented as: % (N).

P value: Non-parametric trend tests for categorical variables, and univariable generalized linear models with polynomial contrasts for continuous variables were used to examine trends across categories.

Table S3. Predictive value of NGAL and Creatinine for alternate WRF

WRF definition	Baseline Values<br>Creatinine AUC	NGAL AUC	P
≥ 0.6 mg/dL increase	0.637	0.656	0.429
≥ 1.2 mg/dL increase	0.678	0.689	0.753
≥ 25% increase	0.575	0.512	0.077
≥ 50% increase	0.515	0.564	0.395
≥ 100% increase	0.524	0.590	0.083
≥ 0.3 mg/dL & ≥ 25% increase	0.532	0.545	0.710
**WRF definition**	**Day 2 values<br>Creatinine AUC**	**NGAL AUC**	**P**
≥ 0.6 mg/dL increase	0.701	0.637	0.142
≥ 1.2 mg/dL increase	0.758	0.672	0.202
≥ 25% increase	0.517	0.512	0.870
≥ 50% increase	0.583	0.564	0.818
≥ 100% increase	0.659	0.590	0.509
≥ 0.3 mg/dL & ≥ 25% increase	0.517	0.520	0.939
**WRF definition**	**Change on day 2<br>Creatinine AUC**	**NGAL AUC**	**P**
≥ 0.6 mg/dL increase	0.768	0.493	<0.001
≥ 1.2 mg/dL increase	0.818	0.486	<0.001
≥ 25% increase	0.737	0.514	<0.001
≥ 50% increase	0.790	0.514	<0.001
≥ 100% increase	0.759	0.574	0.001
≥ 0.3 mg/dL & ≥ 25% increase	0.722	0.506	<0.001

Table S4. Added value of NGAL on top of Creatinine for predicting WRF

WRF definition	MV Model*<br>OR (95% CI)	χ^2	P	AUC	P**
**≥ 0.3 mg/dl increase**					
Creatinine	1.12 (0.98-1.29)	2.87	0.090	0.580	0.013
NGAL	1.18 (1.04-1.35)	6.21	0.013		
**≥ 0.6 mg/dl increase**					
Creatinine	1.33 (1.11-1.59)	9.67	0.002	0.663	0.005
NGAL	1.27 (1.08-1.48)	8.86	0.003		
**≥ 1.2 mg/dl increase**					
Creatinine	1.49 (1.15-1.92)	9.65	0.002	0.705	0.022
NGAL	1.31 (1.05-1.59)	7.16	0.007		
**≥ 25% increase**					
Creatinine	0.69 (0.58-0.82)	16.47	0.000	0.595	0.009
NGAL	1.21 (1.05-1.4)	7.03	0.008		
**≥ 50% increase**					
Creatinine	0.84 (0.65-1.07)	1.83	0.176	0.603	0.013
NGAL	1.27 (1.06-1.51)	7.51	0.006		
**≥ 100% increase**					
Creatinine	1.01 (0.67-1.44)	0.00	0.953	0.603	0.058
NGAL	1.31 (0.99-1.61)	5.34	0.021		
**≥ 0.3 mg/dL & ≥ 25% increase**					
Creatinine	0.8 (0.67-0.94)	6.66	0.010	0.576	0.007
NGAL	1.22 (1.06-1.42)	7.69	0.006		

Figure S1. Changes in serum Creatinine and NGAL in patients with and without WRF, defined as a creatinine increase of ≥ 25%

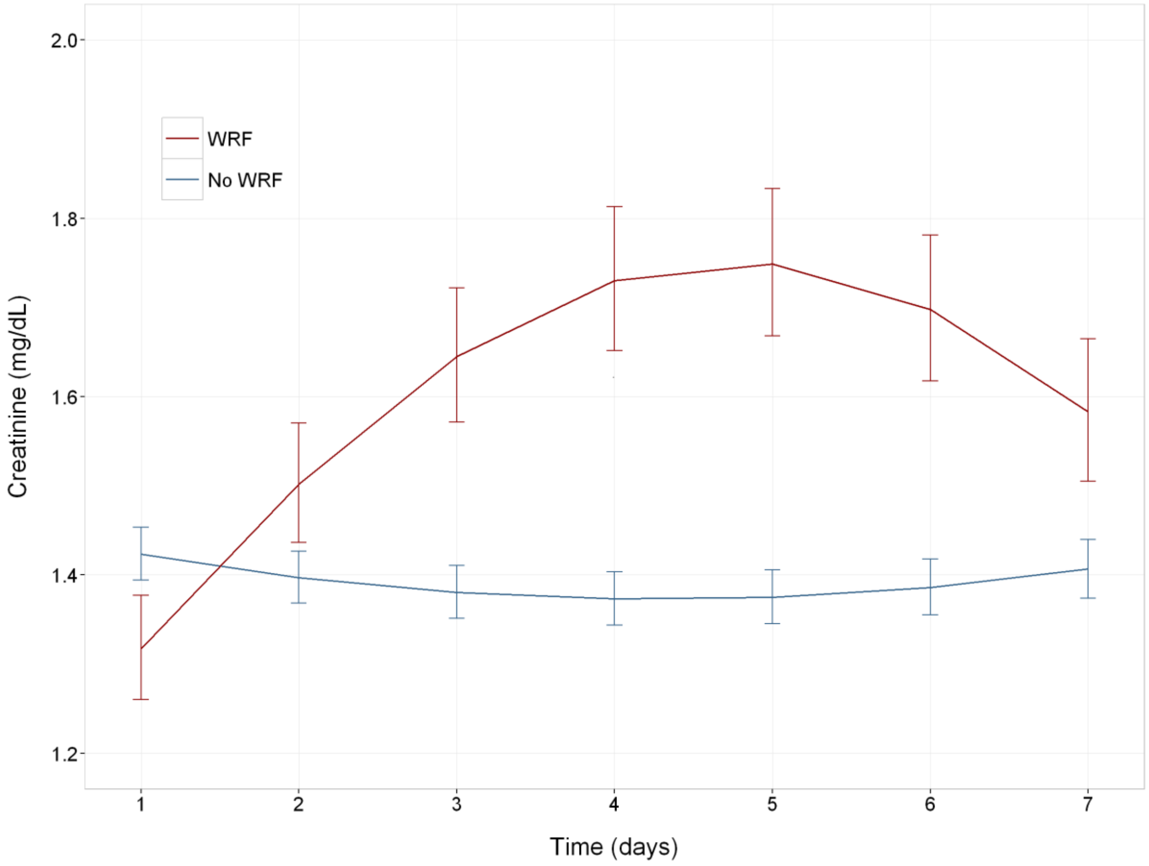

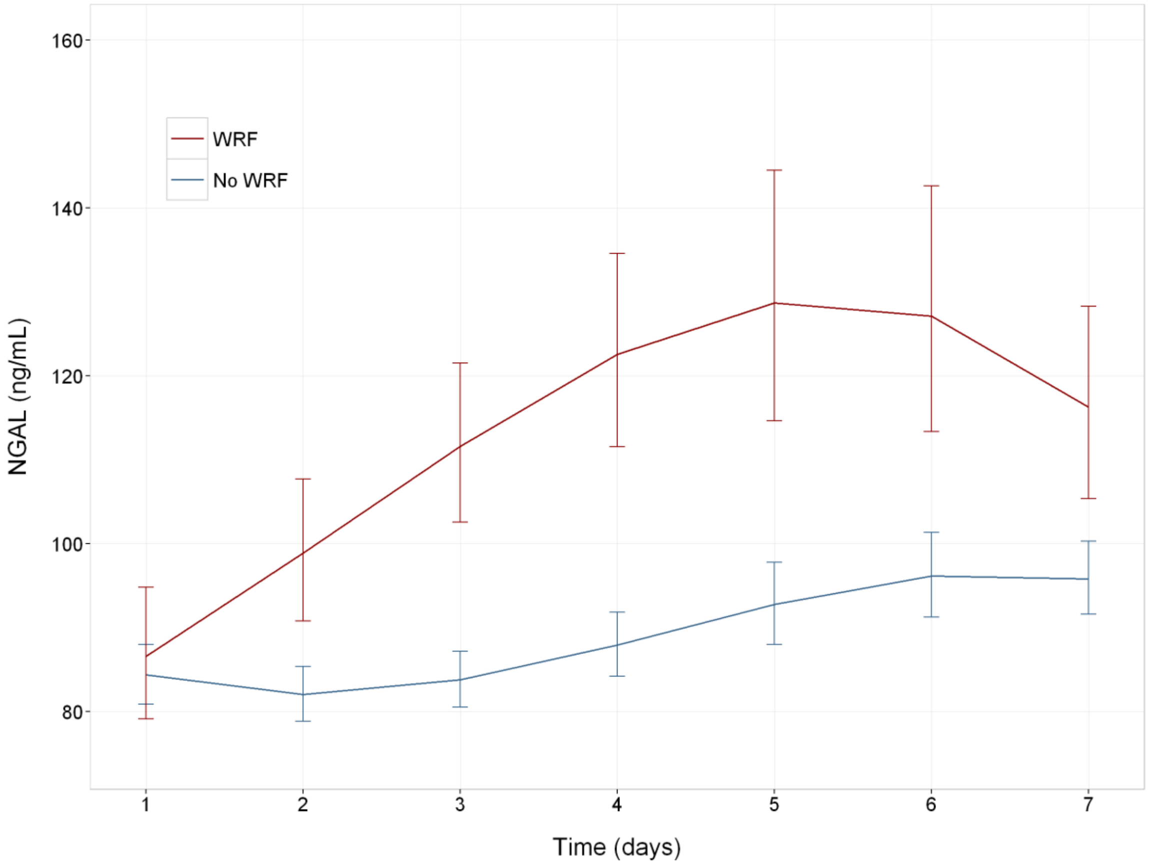

Least square means with 95% confidence intervals
WRF: worsening renal function, defined as creatinine rise ≥ 25% by day 4

Figure S2. Changes in serum Creatinine and NGAL in patients with and without WRF, defined as a combined creatinine increase of ≥0.3 mg/dL and ≥ 25%

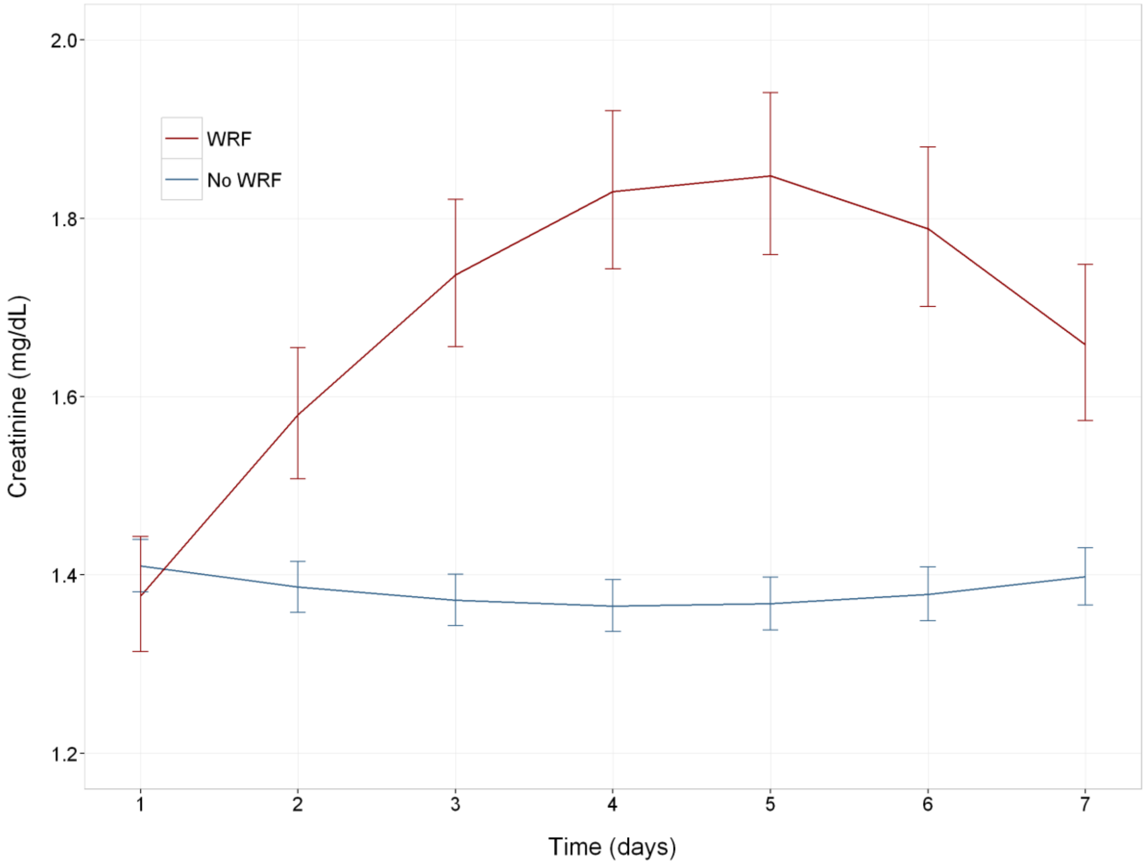

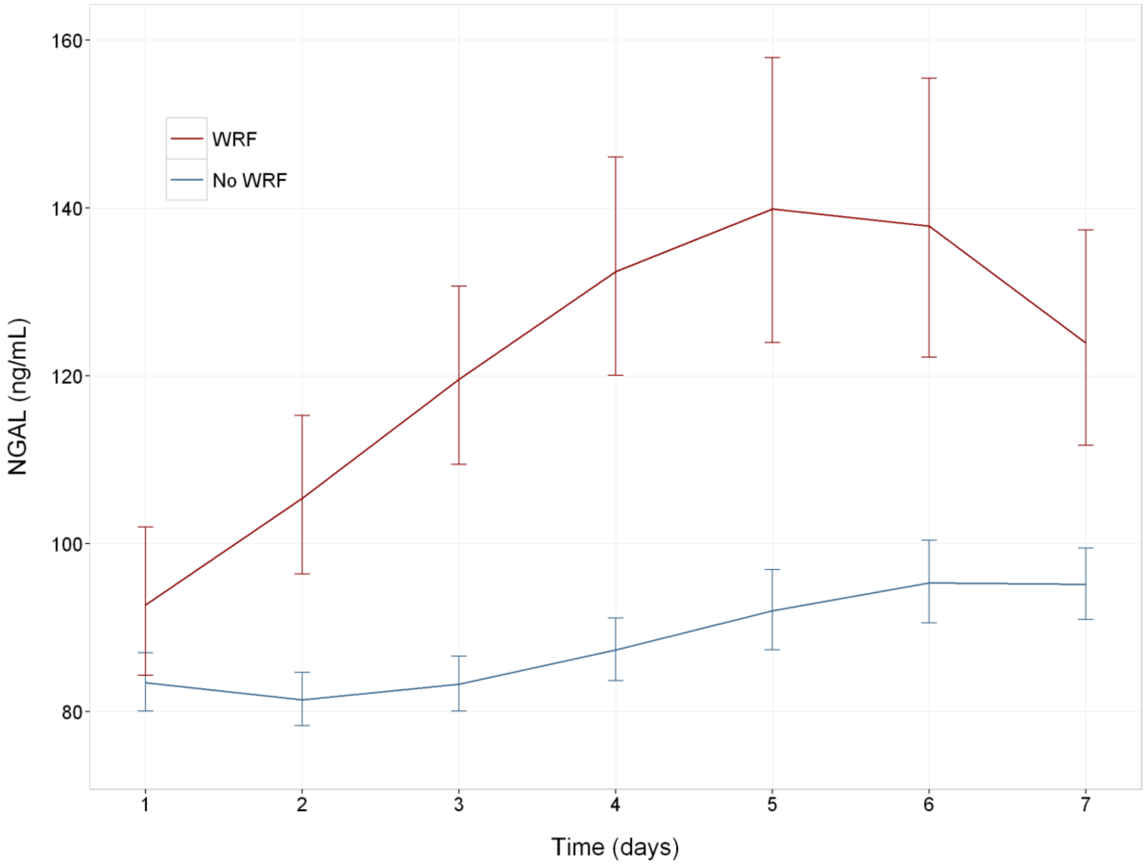

Least square means with 95% confidence intervals over time

WRF: worsening renal function, defined as combined creatinine increase of ≥ 0.3 mg/dL and ≥25% by day 4

Figure S3. Relative changes in serum Creatinine and NGAL in patients with WRF, defined as a creatinine increase of ≥ 25%

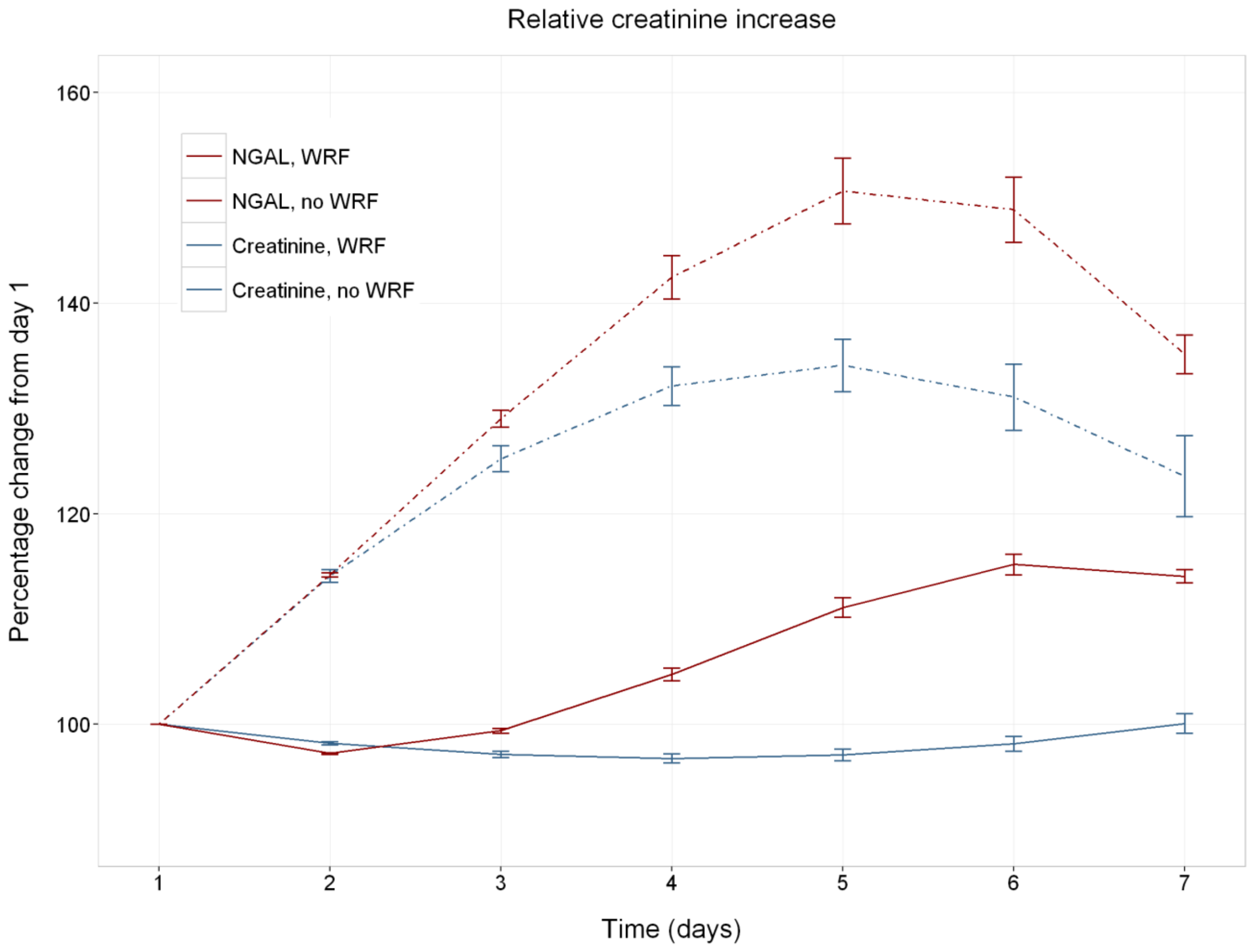

Figure S4. Relative changes in serum Creatinine and NGAL in patients with WRF, defined as a combined creatinine increase of ≥0.3 mg/dL and ≥ 25%

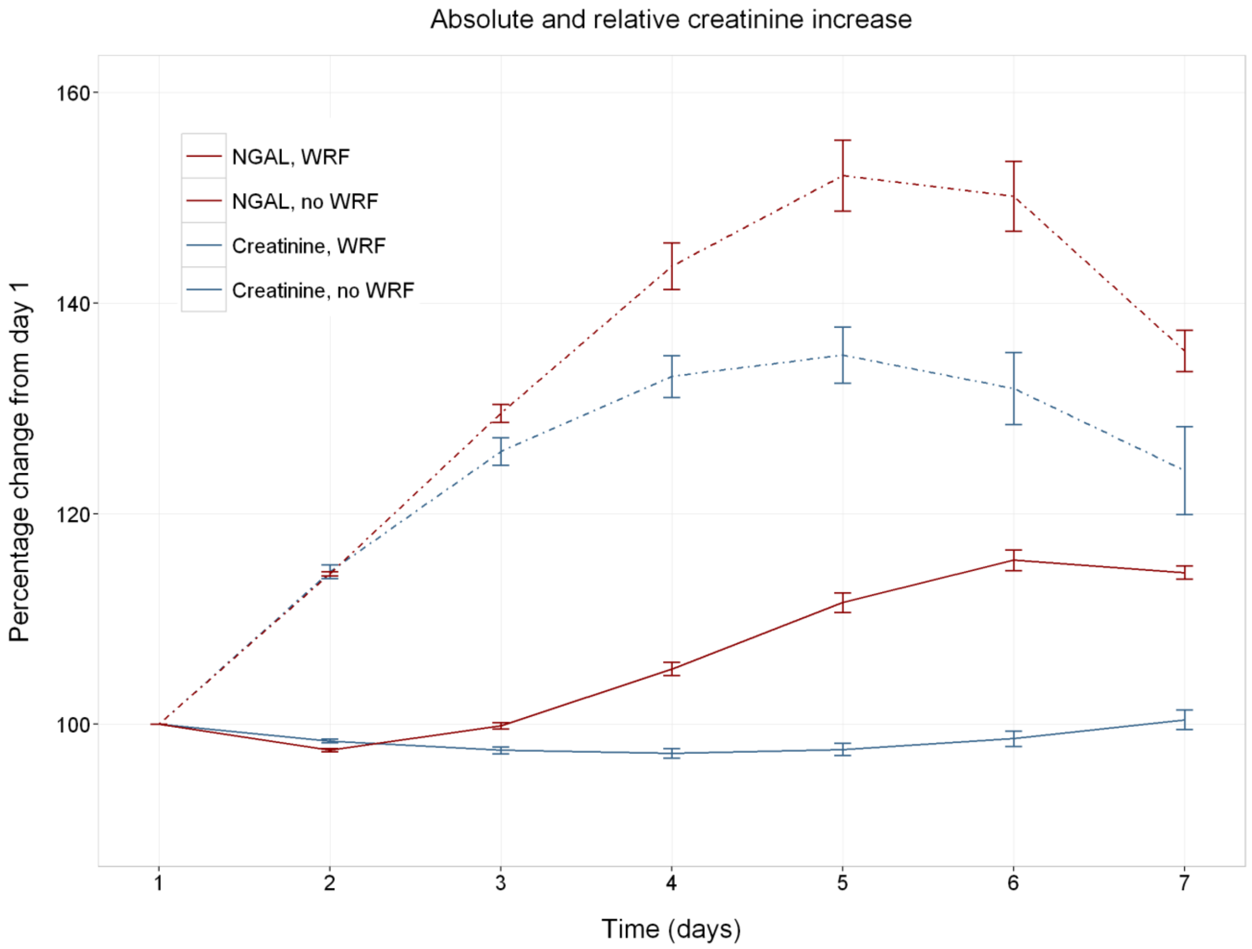

Figure S5. Relationships of baseline NGAL, creatinine and absolute creatinine change with 180-day mortality and the 60-day composite endpoint

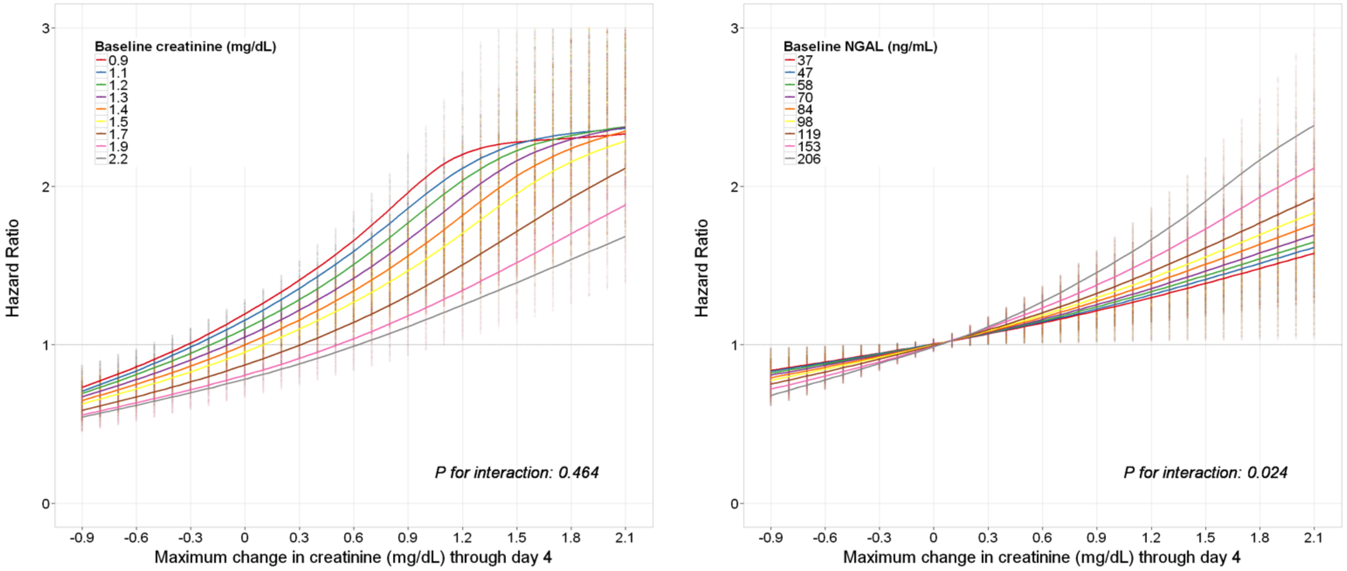

Supplement: Supplementary file 1 [file ijms-18-01470-s001.pdf]
